# GATA1 Activity Governed by Configurations of *cis*-Acting Elements

**DOI:** 10.3389/fonc.2016.00269

**Published:** 2017-01-09

**Authors:** Atsushi Hasegawa, Ritsuko Shimizu

**Affiliations:** ^1^Department of Molecular Hematology, Tohoku University Graduate School of Medicine, Sendai, Japan; ^2^Department of Molecular Oncology, Institute of Development, Aging and Cancer, Tohoku University, Sendai, Japan; ^3^Medical Mega-Bank Organization, Tohoku University, Sendai, Japan

**Keywords:** *cis*-acting elements, GATA1 transcription factor, DNA-binding domain, protein–protein interactions, erythropoiesis

## Abstract

The transcription factor GATA1 regulates the expression of essential erythroid and megakaryocytic differentiation genes through binding to the DNA consensus sequence WGATAR. The GATA1 protein has four functional domains, including two centrally located zinc-finger domains and two transactivation domains at the N- and C-termini. These functional domains play characteristic roles in the elaborate regulation of diversified GATA1 target genes, each of which exhibits a unique expression profile. Three types of GATA1-related hematological malignancies have been reported. One is a structural mutation in the GATA1 gene, resulting in the production of a short form of GATA1 that lacks the N-terminal transactivation domain and is found in Down syndrome-related acute megakaryocytic leukemia. The other two are *cis*-acting regulatory mutations affecting expression of the *Gata1* gene, which have been shown to cause acute erythroblastic leukemia and myelofibrosis in mice. Therefore, imbalanced gene regulation caused by qualitative and quantitative changes in GATA1 is thought to be involved in specific hematological disease pathogenesis. In the present review, we discuss recent advances in understanding the mechanisms of differential transcriptional regulation by GATA1 during erythroid differentiation, with special reference to the binding kinetics of GATA1 at conformation-specific binding sites.

## Introduction

GATA1 is an essential transcription factor (TF) in erythroid and megakaryocyte differentiation that regulates a considerable number of target genes involved in the proliferation, differentiation, and survival of hematopoietic progenitors. GATA1 is a member of GATA factor family, which recognizes the GATA-binding (T/A)GATA(A/G) motif (1). Six members are found in vertebrates and are divided into two subfamilies: GATA1/2/3 belong to the hematopoietic subfamily (2), while GATA4/5/6 are referred to as the endodermal GATA factors (3). GATA1 is expressed in lineage-committed progenitors preprogrammed toward erythrocytes, megakaryocytes, eosinophils, and mast cells (4), whereas GATA2 is abundantly expressed in hematopoietic stem cells, early multipotent progenitors, and monocyte-lineage-committed cells (5, 6). GATA1 and GATA2 display partially overlapping expression patterns during erythroid and megakaryocytic differentiation (7, 8).

Expression of the *Gata1* gene in hematopoietic stem cells and early progenitor cells is repressed by an epigenetic mechanism that precludes access of GATA2 (9). Along with differentiation, GATA2 primes *Gata1* gene expression and results in *Gata1* gene self-activation and *Gata2* gene repression by GATA1. Consequently, GATA1 and GATA2 expression levels show dynamic features, referred to as “GATA-factor switching,” during erythroid differentiation (10). GATA1 and GATA2 partially share binding sites at target genes, acting both redundantly and competitively to regulate gene expression (11). Therefore, the balance of GATA1 and GATA2 expression contributes to fine-tuning the transcriptional regulation of target genes.

GATA1 has four functional domains, consisting of two transactivation domains that reside in the amino (N)- and carboxyl (C)-termini and two zinc-finger domains that are referred to as N- and C-terminal zinc fingers in the middle of the protein. The former two function redundantly and cooperatively to transcriptionally regulate individual target genes (12), while the latter two are highly conserved in all GATA factors as DNA-binding and interaction domains for regulatory proteins and other TFs. The C-finger domain (CF) is particularly indispensable for DNA binding by GATA1, while the N-finger domain (NF) is insufficient for DNA binding alone but stabilizes DNA binding by the CF (13, 14). The NF is especially important for Friend of GATA1 (FOG1) interaction (15) and GATA1 homodimerization (16–19). Multiple molecules have been identified to interact with the CF, such as LIM domain only 2 (LMO2) (20) and MED1, a component of the mediator complex (21). Thus, a dual zinc-finger structure appears to modulate DNA-binding and protein–protein interactions to form the characteristic GATA1 complex and leads to the diverse target gene expression regulation.

Several hematopoietic disorders are linked to GATA1 dysfunction. Germ-line and somatic *GATA1* gene mutations that produce a short form of GATA that lacks the N-terminal transactivation domain are causal in Diamond–Blackfan anemia (22) and preleukemic disease in Down syndrome patients (23), respectively. Substitution mutations in the NF are associated with X-linked hematopoietic diseases and are accompanied by thrombocytopenia, porphyria, and dyserythropoietic anemia (24). Cell-based complementation approaches have determined how the mutations alter the functions of GATA1 (25). Furthermore, genetically manipulated mouse models that phenocopy human diseases have been established and provide insight into the pathogenesis caused by GATA1 dysfunction (26–29). Furthermore, quantitative reduction of GATA1 has been described as causal in acute erythroblastic leukemia and myelofibrosis in mice (30–32).

GATA-binding motifs are found scattered in a variety of genes that are distributed throughout the genome. However, it is largely unknown how GATA1 properly organizes diversified gene expression to generate distinct expression profiles during erythropoiesis. Recent comprehensive analyses of GATA1 occupancy have shown that diversities in the neighboring sequence of consensus GATA-binding motifs can modify the transcriptional output. Multiple motifs of transcription regulation may act in a synergistic manner when they align in order with the GATA-binding motif in a *cis*-acting element. In this article, we describe diverse DNA binding and transcriptional regulation mediated by GATA1 during erythropoiesis, focusing on the *cis*-acting element configuration.

## Composite Elements with an E-Box and a GATA-Binding Motif

**Figure 1:**
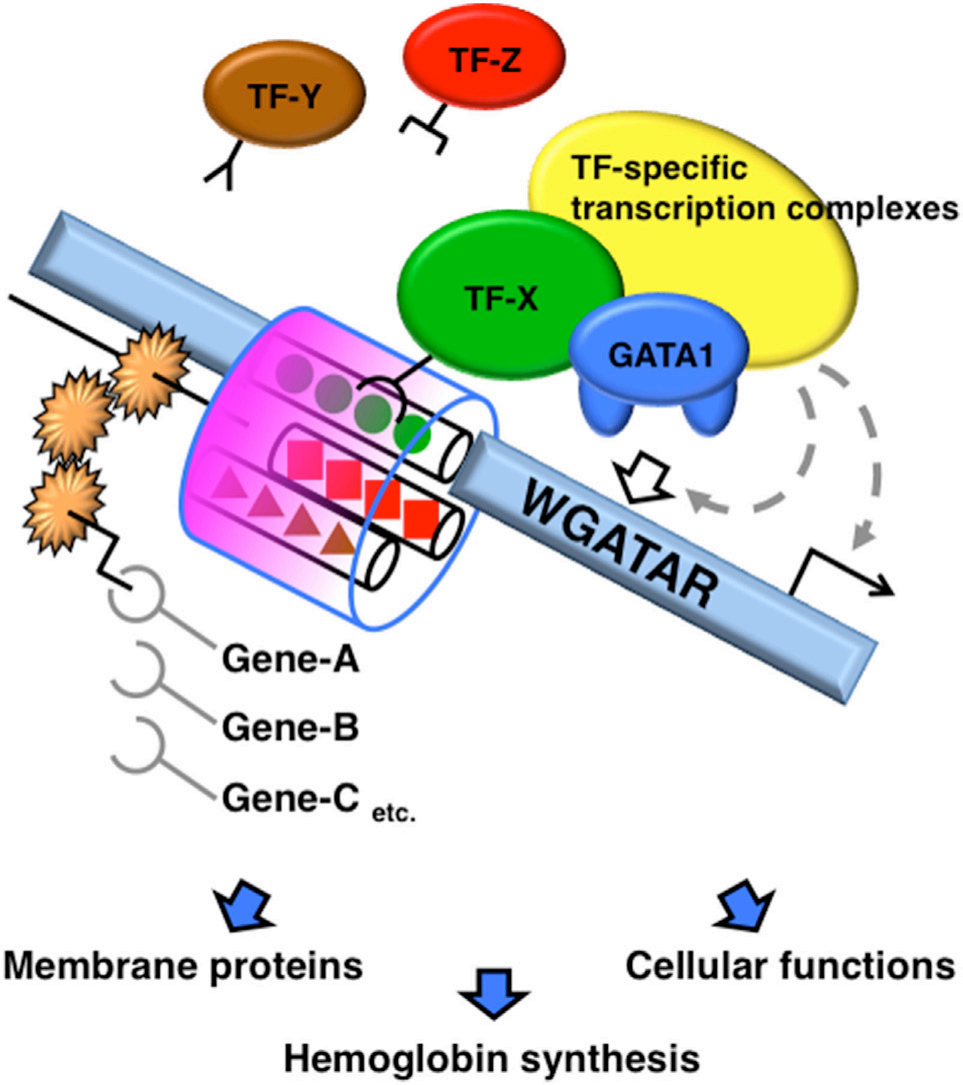
**Schematic diagram of GATA1 functional modifications mediated by sequences adjacent to the GATA-binding site**. A transcription factor (TF) recruited to the region adjacent to the GATA-binding motifs modifies the DNA binding, kinetics, and stoichiometry of GATA1. Consequently, the GATA1-centered transcriptional complex formation on the *cis*-acting elements is altered, which alters the transcriptional activity of GATA1.

A number of molecules reportedly interact with GATA1 and modulate its function. TFs recruited with GATA1 diversify the transcriptional output based on how the molecules complex with GATA1. Accretion of TFs to the neighboring regions of the GATA-binding motif appears to modify the DNA binding, kinetics, and stoichiometry of GATA1, and subsequently modifies the formation of GATA1-centered transcriptional complexes on the *cis*-acting elements (Figure 1).

Transcription factors that interact with GATA1 have specific motifs that are enriched in repetitive regions of the genome. Chromatin immunoprecipitation (ChIP)-sequencing analyses identified GATA1 (20, 33–35) and GATA2 (36) interactions and co-localization with SCL on the E-box–GATA combined motif, thus forming a large protein complex with E2A, LMO2, and LIM domain-binding 1. SCL is a member of the basic helix-loop-helix family of TFs, which recognize DNA-binding sites containing the E-box motif (CANNTG) as a heterodimer with E2A (37). The E-box sequence is enriched 7–12 bp upstream of GATA motifs in erythroid-specific genes. The DNA-binding affinities of GATA1 and SCL to the combined E-box–GATA motif are increased by co-localization with each other, thereby modifying the transcriptional activity of GATA1 (37–39). SCL occupancy is often found at GATA-occupied loci lacking an E-box (40). By contrast, SCL occupancy is abrogated by the lack of GATA1 on the E-box–GATA motif in certain genes, such as genes encoding erythroid-specific 5-aminolevulinate synthase, uroporphyrinogen III synthase, and pyruvate kinase, in which single-nucleotide mutations of the GATA motif have been found in patients with erythroid disorders (41). Thus, the combination of GATA-binding and E-box motifs contributes to specific transcriptional regulation by varying the DNA-binding characteristics and structure of the complex containing GATA1 and SCL, and possibly by varying other components of the complex (Figure 1).

The large complex binding to the E-box–GATA motif usually acts as a transcription activator (40). Interestingly, in the genes activated by GATA2 and repressed by GATA1, SCL is co-localized with GATA2 when the gene is activated, while the SCL occupancy is decreased when GATA1 takes the place of GATA2 (40). One plausible explanation for the difference in SCL occupancy is that differences in undesignated sequences in the composite E-box–GATA motif may influence complex formation, depending on whether GATA1 or GATA2 is present. Binding analyses utilizing naked DNA have indicated that SCL preferentially binds to CAGGTG (33) or CAGATG (42) motifs in essential hematopoiesis genes, whereas CATCTG sequences are enriched in GATA2-occupied loci in lineage-negative hematopoietic progenitors (43). Furthermore, the spacer lengths and sequences between E-box and GATA-binding motif vary at individual loci. We envision delicate differences in sequence alignment among E-box–GATA composite elements conferring changes in complex structure, DNA-binding affinity, and protein–protein interactions, consequently diversifying target gene expression profiles.

## CACC-Box Adjacent to GATA-Binding Motif

Genome-wide ChIP-sequencing analyses have revealed that the NCNCNCCCN (extended CACC) motif is frequently found in the region of GATA1 ChIP peaks in erythroid-committed cells (44, 45). The CACC motif is a known consensus sequence recognized by Krüppel-like transcription factors (KLFs). Among the 17 mammalian KLFs, KLF1 (EKLF), KLF2, and KLF13 are involved in erythrocyte maturation and differentiation (46–48). In particular, the quantitative ratio of GATA1 and KLF1 in the nucleus is tuned by their acetylation states, which are mediated by HDACs, to control erythroid-specific gene expression (49–51). Many KLF1 variants have been found in recent years as the causes of different types of red cell disorders (52, 53).

Krüppel-like transcription factors have three conserved C2H2-type zinc fingers at the C-terminus that are involved in DNA binding and directly interact with GATA1 (54). When the GATA and CACC motifs are located close together in the regulatory region, GATA1 and KLF1 improve the DNA-binding affinity of each other, synergistically increasing gene expression in hematopoietic and non-hematopoietic cells (54, 55). Thus, a composite element composed of GATA- and CACC-binding motifs works as a *cis*-regulatory region distinct from each individual motif’s function (Figure 1). ChIP-sequencing peaks of GATA1 and KLF1 overlap in several gene loci. However, the number of co-occupied loci is lower than expected based on the significant cooperative function of GATA1 and KLF1 in the erythroid differentiation (56, 57). One plausible explanation is that other KLFs share CACC motif binding with KLF1 when adjacent to GATA motif. In addition, TFs belonging to specificity protein (SP) family bind to the CACC motif and have a triple-C2H2-type DNA-binding domain highly conserved with KLFs. SP1, which has relatively ubiquitous expression pattern and plays key roles in critical biological process through regulating metabolic genes (58), is reported to interact and cooperatively work with GATA1 to control gene expression (54, 59). Furthermore, ZBP-89, a Krüppel-type zinc finger TF, has a potential to bind CACC motifs and complex with GATA1 (60). KLF2 regulates embryonic erythropoiesis through redundantly and cooperatively working with KLF1 (47, 61). Therefore, other TFs that share CACC motif binding with KLF1 may influence KLF1 function and consequently influence GATA1 function (Figure 1). Questions regarding how the undesignated nucleotides in the consensus CACC motif contribute to the preferential binding of KLFs and SPs remain to be answered, and GATA1 function may be modified by alternate factors recruited to the CACC motif.

## Binding Motifs for Collaborative TFs

In addition to the E-box and CACC motifs, a variety of *cis*-regulatory sequences found adjacent to the GATA-binding motif have been reported to mediate the transcriptional output of GATA1 during erythroid differentiation. Consensus NFE2-binding (C/T)GCTGA(C/G)TCA(C/T) motifs are found close to the GATA-binding motif in genes encoding β-globin and erythroid-specific membrane protein (62, 63). CP2 was originally identified as a regulator for human α-globin gene expression (64) and regulates erythroid differentiation through the binding at the CNRG-N_5-6_-CNR(G/C) motif. A CP2-binding motif adjacent to the GATA motif is required in the regulation of erythroid-specific genes, such as mouse *Gata1*, mouse *Klf1*, mouse *Nfe2*, mouse *Epor*, human/mouse *UROS*, the human/mouse globin gene clusters, and human *CDC6, in vivo* (65–72).

We assume that the sequence adjacent to the GATA motif may alter the DNA-binding mode of GATA1, the formation of transcription complexes, and organization of chromatin structures if a TF recognizes and binds the sequence. Given these associations, we propose that sequence alignments of regions neighboring the GATA-binding motifs influence the transcriptional activity of GATA1 (Figure 1).

## Composite Elements with Dual GATA1-Binding Motifs

We noticed that one or more GATA-binding motifs are found in scattered regions throughout the genes involved in erythropoiesis. Given that the sequence adjacent to the GATA-binding motif is varied in each region, individual GATA1 is predicted to access *cis*-regulatory regions differentially to contribute to gene expression and to allow sophisticated gene regulation. *cis*-Targeting experiments have found that each GATA-binding motif independently and redundantly plays a role in the expression of certain genes in mice (65, 66, 73, 74). However, the regulation of the spatiotemporal control of gene expression is poorly understood.

Focusing on the GATA-binding motif, one significant observation is that two or more GATA-binding motifs are sometimes oriented closely in line, generating composite GATA elements in which GATA-binding motifs are aligned side-by-side in either tandem or palindromic orientation. Considering that GATA1 forms a homodimer through NF and CF interactions (19), composite GATA elements composed of two GATA-binding motifs may have a more significant function than two single GATA-binding motifs. Furthermore, the DNA-binding structure of GATA3 revealed that the NF binds the opposite face of DNA that is bound by the CF and interacts with the C-terminal basic-tail of the CF that is inserted into the minor groove (75). Therefore, when two GATA-binding motifs are aligned side-by-side, the direction of the two GATA-binding motifs seems to be important.

## Palindromic Dual GATA-Binding Motifs

When two GATA-binding motifs are aligned in a palindromic orientation, two types of composite GATA elements are generated: either a head-to-head (YTATCW–WGATAR) or tail-to-tail (WGATAR–YTATCW) orientation. The difference in orientation may influence GATA1 binding modes. For simplicity’s sake, we refer to the GATA-binding motifs aligned in head-to-head and tail-to-tail palindromic orientations as Pal-GATA or rPal-GATA motifs, respectively.

GATA1 binds to DNA with the CF while the NF scarcely functions during DNA binding (76). Indeed, the association and dissociation kinetics of GATA1 on a single-GATA motif on naked DNA, as measured in DNA-binding surface plasmon resonance (SPR) studies, do not change regardless of the NF function (77). By contrast, an electrophoretic mobility shift assay showed that the NF can associate with DNA on GAT(N) sequences, although the binding affinity is too weak to support GATA1–DNA binding independently (14). Therefore, in cases where a GAT(N) sequence is aligned adjacent to a GATA-binding motif that is bound by the CF, the NF may contribute to DNA binding. Particularly, the NF increases GATA–DNA-binding affinity in the Pal-GATA motif on both naked and chromatin DNA (14, 78).

There are three important observations about the N-finger function regarding its DNA binding. First, the DNA-binding kinetics at Pal-GATA motifs with R216 substitution mutations are similar to those of GATA1 lacking an entire NF, indicating the R216 residue is essential for NF-DNA association. The R216 residue is located on the opposite face to the FOG1 association face (20). Therefore, NF-DNA binding appears to be independent of FOG1 association. To date, multiple substitution mutations in the NF have been found in inherited human diseases (24, 78). The disease pathogenesis caused by the R216 mutation likely differs from that due to impaired FOG1 association, although the disease phenotypes partially overlap (79–82).

**Figure 2:**
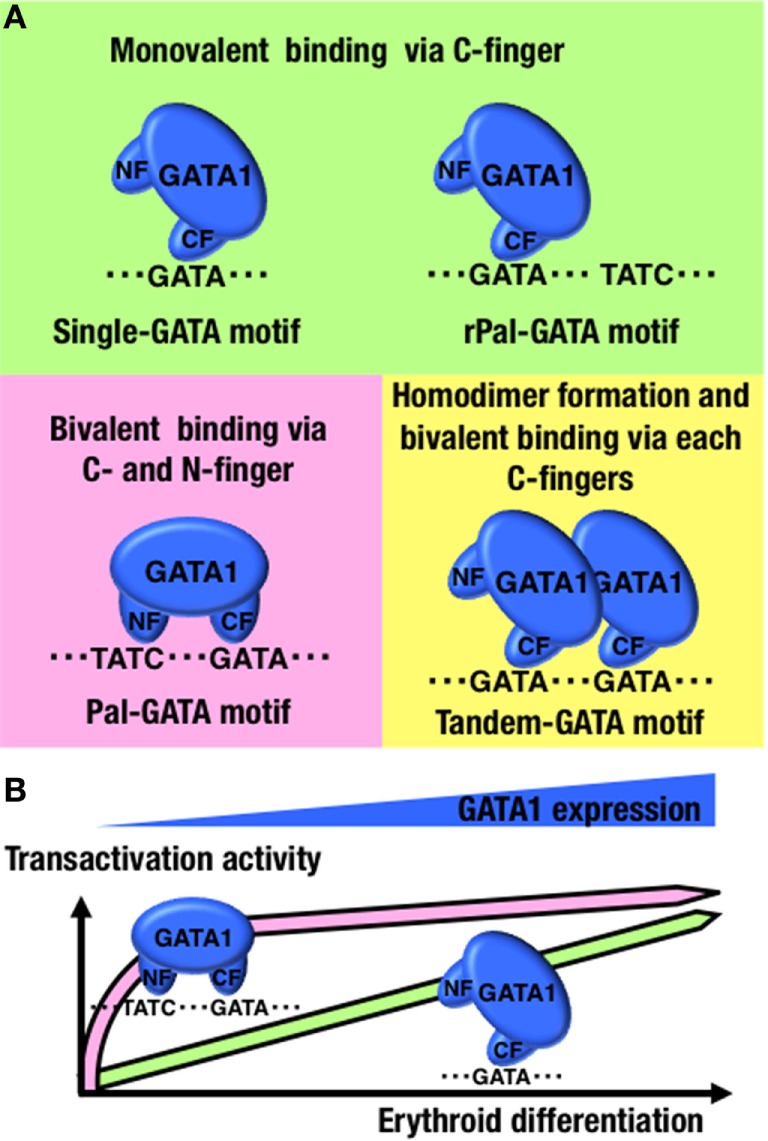
**GATA1-binding kinetics at conformation-specific binding sites**. **(A)** Schematic diagrams of the GATA1-binding modes at the indicated configurations of GATA-binding motifs. **(B)** Differences in transactivation dynamics of GATA1 between single- and Pal-GATA motifs. NF, N-finger domain; CF, C-finger domain.

Second, the NF prefers to bind to a specific configuration of two GATA-binding motifs. SPR analysis showed that GATA1 lacking an NF binds to rPal-GATA motif similarly to wild-type GATA1 (77). GATA1 with a substitution mutation at R216 binds to tandem-oriented GATA motifs (Tandem-GATA motif) similarly to wild-type GATA1 (77), suggesting that the NF-DNA association is not critical for binding to the rPal-GATA motif and Tandem-GATA motif. By contrast, the SPR parameters of GATA1 binding at Pal-GATA motifs do not fit the 1:1 binding model found at single-GATA motifs (77). GATA1 binds to single-GATA motifs monovalently through the CF, while Pal-GATA motif binding is bivalent and uses both the CF and NF (Figure 2A). Thus, GATA1 differentially binds single-GATA and Pal-GATA motifs.

Lastly and importantly, if the NF loses its DNA-binding capacity, the disabled GATA1 binds to Pal-GATA motifs monovalently, as it would at single-GATA motifs. Regardless of the NF function in DNA binding, GATA1 is able to bind to any configuration of GATA-binding motifs, although the GATA1 occupancy levels at Pal-GATA motifs depend on the binding capacity of the NF (77). Therefore, monovalent binding of GATA1 to the Pal-GATA motif is proposed to retain some activity, though not comparable to the full activity from bivalent GATA1 binding. Importantly, time-course reporter analyses in which GATA1 was introduced into non-hematopoietic cells have revealed that bivalent binding of GATA1 at Pal-GATA motifs is required for transactivation during early phases of GATA1 induction or at low GATA1 expression levels (Figure 2B) (77). GATA1 shows a dynamic expression profile during erythroid differentiation (10). The bivalent binding of GATA1 to Pal-GATA motifs may induce allosteric binding effects and enable GATA1 to precipitously induce target genes during the early erythroid differentiation phases (Figure 1).

## Double GATA Motifs Oriented in Tandem

Similar to GATA1 binding at Pal-GATA motifs, SPR parameters of GATA1 binding at Tandem-GATA motifs does not fit the 1:1 binding model, and the SPR values are not altered by substitution mutations at R216 (77). Furthermore, if GATA1 lacks homodimerization capacities, then the disabled GATA1 binds to Tandem-GATA motifs similarly to GATA1 binding at single-GATA motifs (77). This finding generates an image of two GATA1 molecules in a dimer formation bivalently binding to two tandem-oriented GATA-binding motifs through the individual CFs (Figure 2A). Considering that GATA1 recognizes and binds to single-GATA motifs through the CF, there might be cases in which two GATA1 molecule monomers bind to two GATA-binding motifs aligned in any configuration if GATA1 is abundant, as described in previous studies of transgenic zebrafish (18). The secondary GATA1 molecule more efficiently binds to Tandem-GATA motifs than other configurations of dual GATA-binding motifs by forming a GATA1 homodimer.

In mice and zebrafish, GATA1 dimerization is important for erythropoiesis because it regulates specific genes, including the *Gata1* gene (18, 19). Similar to the Pal-GATA motifs, the GATA1 monomer binds to Tandem-GATA motifs monovalently if GATA1 fails to form a homodimer (77). Taking into consideration the above-mentioned issues, bivalent binding of GATA1 homodimers to Tandem-GATA motifs might have a specific function that differs from the monovalent GATA1 monomers binding at Tandem-GATA motifs (Figure 1).

## Conclusion

Accession to DNA *via* the consensus GATA-binding motif is a fundamental issue in the role of GATA1 as a TF. However, simple GATA1–DNA interactions only explain certain aspects of transcriptional regulation and fail to address comprehensive GATA1 regulation of various target genes, each of which has its own expression profile. Recent findings have made great advances in our knowledge of how the molecules recruited to regions adjacent to GATA-binding motifs modify the binding kinetics, reaction stoichiometry, and complex formations centered with GATA1, which may allosterically regulate GATA1 transcriptional activity (Figure 1). Intriguingly, a feature of GATA1 being a dual zinc-finger structure allows bivalent GATA1 binding to two GATA-binding motifs aligned side-by-side in both palindromic and tandem orientations. This variety may allow further functional diversity in GATA1 binding above monovalent binding for simple GATA-binding motifs. The molecular mechanisms of GATA1 transcriptional activity have been dissected in recent decades, and the contributions of GATA1 mutations to disease pathogenesis have been vigorously investigated. Nonetheless, how the configuration of *cis*-acting elements in the sequence surrounding GATA-binding motifs can generate diversified GATA1 transcriptional activity remains to be explored.

## Author Contributions

All the authors listed have made substantial, direct, and intellectual contribution to the work and approved it for publication.

## Conflict of Interest Statement

The authors declare that the research was conducted in the absence of any commercial or financial relationships that could be construed as a potential conflict of interest.
